# Novel viruses of *Haloquadratum walsbyi* expand the known archaeal virosphere of hypersaline environments

**DOI:** 10.1093/ismejo/wraf149

**Published:** 2025-07-17

**Authors:** Judith Villamor, María Dolores Ramos-Barbero, Mercedes Moreno-Paz, Cristian Villena-Alemany, Manuel Martínez-García, Víctor Parro, Josefa Antón, Fernando Santos

**Affiliations:** Department of Physiology, Genetics, and Microbiology, University of Alicante, San Vicente del Raspeig 03690, Alicante, Spain; Department of Physiology, Genetics, and Microbiology, University of Alicante, San Vicente del Raspeig 03690, Alicante, Spain; Departament de Genètica, Microbiologia i Estadística, Universitat de Barcelona, Diagonal 643. Annex. Floor 0, Barcelona E-08028, Barcelona, Spain; Department of Molecular Evolution, Centro de Astrobiología (CAB), INTA-CSIC, Torrejón de Ardoz 28850, Madrid, Spain; Department of Physiology, Genetics, and Microbiology, University of Alicante, San Vicente del Raspeig 03690, Alicante, Spain; Centre Algatech, Institute of Microbiology, Czech Academy of Sciences, 379 01 Třeboň, Czech Republic; Department of Physiology, Genetics, and Microbiology, University of Alicante, San Vicente del Raspeig 03690, Alicante, Spain; Multidisciplinary Institute of Environmental Studies Ramon Margalef, University of Alicante, San Vicente del Raspeig 03690, Alicante, Spain; Departament de Genètica, Microbiologia i Estadística, Universitat de Barcelona, Diagonal 643. Annex. Floor 0, Barcelona E-08028, Barcelona, Spain; Department of Physiology, Genetics, and Microbiology, University of Alicante, San Vicente del Raspeig 03690, Alicante, Spain; Multidisciplinary Institute of Environmental Studies Ramon Margalef, University of Alicante, San Vicente del Raspeig 03690, Alicante, Spain; Applied Biomedicine Group (Alicante Institute for Health and Biomedical Research, ISABIAL), Alicante 03080, Alicante, Spain; Department of Physiology, Genetics, and Microbiology, University of Alicante, San Vicente del Raspeig 03690, Alicante, Spain; Applied Biomedicine Group (Alicante Institute for Health and Biomedical Research, ISABIAL), Alicante 03080, Alicante, Spain

**Keywords:** Haloquadratum, hypersaline, virome, halovirus, halophile, archaeal virus

## Abstract

Solar salterns represent unique systems with low diversity microbial communities that serve as an excellent model for studying the evolution and ecology of archaeal viruses and the interactions with their hosts. This is particularly relevant for the extremely abundant “square” archaeon *Haloquadratum walsbyi*, for which isolated viruses have remained elusive despite the fact that this microbe governs the salt-saturated ponds of most solar salterns worldwide. In this work, we have used cutting-edge imaging techniques, based on virus fluorescence in situ hybridization (virusFISH), and a combination of -omic techniques, at both population and single-cell levels, to provide an in-depth characterization of the *Hqr. walsbyi* virosphere. Our analyses have led to the identification of a new subfamily of tailed low-GC dsDNA viruses, which we propose to name “Haloquadravirinae”, with host assignment confirmed by virusFISH in natural samples. Haloquadraviruses can represent more than 50% of the viral community in solar saltern viromes and infect nearly 40% of square cells in natural environments. The genetic imprint of these viruses, which are globally distributed in hypersaline environments, has provided insights into the structure of their virions and their potential life strategy. Along with the identification of other virus-like elements associated with *Hqr. walsbyi* through single-cell genomics, this work expands our current understanding of the archaeal virosphere.

## Introduction

Our knowledge about the archaeal virosphere, although still very limited, is gradually growing thanks to culture-independent strategies, in particular, viral metagenomics [1]. This is especially relevant for uncultured archaeal lineages that play important roles in nature, such as thaumarchaea, Asgard archaea, methanotrophic archaea, or the marine group *Candidatus* Poseidoniales, some of which have been recently linked to viruses through analyses of viromes [2–5]. In the case of halophilic archaea, around 100 viruses have been isolated so far [6–10]. However, most of them infect easy-to-grow hosts which are not frequently abundant in hypersaline environments.

Among the hypersaline systems, the microbial communities of the multi-pond solar salterns have been extensively studied [11, 12]. These environments consist of circuits of interconnected ponds of increasing salinity ending in the crystallizers, where sodium chloride precipitates and is harvested for commercial use. In salterns, where salinity increases, low diversity and archaea-dominated communities reach high densities. Viruses infecting halophilic microbes, named haloviruses, increase as well, reaching the highest concentrations of virus-like particles (VLPs) described in aquatic systems [13, 14]. Therefore, solar salterns constitute systems with simplified niches that serve as models to study virus-host interactions and to understand their ecology and evolution. This is particularly outstanding if the host species is ecologically relevant, such as the “square” archaeon *Hqr. walsbyi* [15, 16], which dominates in crystallizers reaching up to 80% of the total cells [17]. Although there are numerous studies focused on *Hqr. walsbyi* [15, 18], no isolated viruses have been obtained due to the impossibility of this microbe to form lawns on agar plates for plaque assays. So far, the only evidence of square cells infected by viruses, reported decades ago, relied on transmission electron microscopy of hypersaline brine samples [19, 20].

Genomics revealed the first virus-related sequences within the chromosomes of *Hqr. walsbyi* type strains [21]. Furthermore, the study of PL6-family plasmids—present in some members of the species—suggested a strong association with viruses, potentially representing a novel group of proviruses [22]. In parallel, culture-independent approaches provided new insights into viruses potentially infecting *Haloquadratum*. The construction of metagenomic libraries from haloviral DNA, directly extracted from the CR30 crystallizer at the Bras del Port salterns (Santa Pola, Alicante, Spain), enabled the identification of the first viral sequences tentatively associated with *Hqr. walsbyi* based on DNA signatures [21, 23–25]. Some of these sequences, derived from fosmid libraries, were classified within cluster 1 of “environmental halophages” (eHPs), a group of viral genomes characterized by a ~44% GC content (“low-GC”) and containing multiple proto-spacers matching the CRISPR systems of *Hqr. walsbyi* C23^T^ [25]. Additional viral sequences, obtained from a shotgun cloning library and exhibiting a ~57% GC content (“intermediate-GC”), also showed strong identity with *Hqr. walsbyi* C23^T^ spacers, distinct from those associated with the eHPs [21].

Here, we have focused on the study of viruses infecting *Haloquadratum* by using a combination of metagenomics, single-cell genomics, and imaging-based technologies. First, a hyperhalophilic viral assemblage was incubated in the presence of *Hqr. walsbyi* pure cultures in order to selectively enhance the retrieval of *Haloquadratum* viruses. Second, the *Haloquadratum*-enriched virome was hybridized against a “virochip” with a collection of haloviral genomes, previously used to identify the first virus of an uncultured member of *Nanohaloarchaeota* [26]. This enabled the characterization of a new subfamily of *Haloquadratum* viruses, which was later confirmed by virus fluorescence *in situ* hybridization (virusFISH). Subsequently, single-cell genomics, successfully used for host-virus assignments [27–33], was used here in combination with the abovementioned virochip to directly pinpoint infected *Haloquadratum* cells in nature. Overall, this work offers a multiphasic -omic approach for the study of uncultured viruses, while broadening our knowledge about the haloarchaeal virosphere.

## Materials and methods

### *Haloquadratum*-enriched virome

A hypersaline water sample with 36% salinity (measured *in situ* with a hand refractometer, Eclipse) and ~10^7^ cells/ml (measured by DAPI staining, as in Antón et al. [34], with ~20% of square cells), was taken from the CR30 crystallizer of Bras del Port solar salterns (Santa Pola, Alicante, Spain) in February 2014. Its viral community was characterized by transmission electron microscopy (TEM) and metagenomics [35]. In parallel to the work developed in [35], 300 milliliters of the sample (referred to as CR30feb14) were centrifuged (30 000 *xg*, 30 minutes, 20°C, in an Avanti J-30I Beckman centrifuge with a JA rotor) and the supernatant filtered through a 0.2 μm filter to remove cells. One milliliter of the filtered volume, containing the CR30feb14 viral assemblage, was used to determine the abundance of VLPs by Sybr-Gold staining (as in [36]). The rest was enriched with sterilized nutrients (1% pyruvate and 0.05% yeast extract) and then inoculated with *Hqr. walsbyi* (referred to as HQR sample)*.* For the *Hqr. walsbyi* inoculum preparation, the strains DSM 16790 (HBSQ001) and DSM 16854 (C23^T^) were separately grown at 37°C (100 rpm) in DSMZ medium #1091 (containing: 195 g NaCl, 50 g MgSO_4_ · 7H_2_O, 35 g MgCl_2_ · 6 H_2_O, 5 g KCl, 0.25 g NaHCO_3_, 1 g NaNO_3_, 0.5 g CaCl_2_ · 2 H_2_O, 0.05 g KH_2_PO_4_, 0.03 g NH_4_Cl, 0.05 g yeast extract, 1 g sodium pyruvate, 20 ml Tris buffer 1 M, and 1 liter of distilled water, pH 7.4). Culture cell densities were determined by DAPI staining when they showed turbidity (after ~7 months). Volumes from each culture, containing 1.15 × 10^8^ total cells, were centrifuged (4000 *xg*, 20 minutes, 20°C, in an Avanti J-30I Beckman centrifuge with a JA-14 rotor) and cell pellets resuspended in 5 ml of supernatant. Both concentrates were joined and mixed, and 5 ml were added to the HQR flask, which was incubated at 37°C for 19 days without shaking.

After the incubation, cells in HQR were removed and one milliliter of sample was used for VLP counts and TEM (as in [35]). For viral metagenomics, viruses in HQR were concentrated by tangential flow filtration (through a Vivaflow filter cassette system with a molecular weight cutoff of 30 000 Daltons) and ultracentrifugation (186 000 *xg*, 2 hours, 20°C, in an Optima MAX-XP Ultracentrifuge with a TLA-55 rotor, Beckman Coulter). The virus pellet was resuspended with 200 μl of the corresponding supernatant, mixed with equal volumes of 1.6% low-melting-point agarose (Pronadisa), dispensed into 100 μl molds, and allowed to solidify at 4°C. The plugs were incubated with three units of Turbo DNase (Ambion), for 1 hour at 37°C, to remove contaminant dissolved DNA, and incubated overnight at 50°C in ESP (0.5 M EDTA, pH 9.0, 1% N-laurylsarcosine, and 1 mg/ml proteinase K) to disrupt the viral capsids. HQR viral DNA was extracted as described in Villamor et al. [35], quantified using Qubit (Invitrogen) and sequenced at FISABIO (Valencia, Spain), using a MiSeq System (Illumina) (2x300 bp), to obtain the HQR virome (HQR-V).

### Virochip hybridization and fosmid purification

A microarray (or “virochip”), containing 364 triplicated and immobilized viral fosmid clones from a CR30 water sample taken in 2011 (CR30jun11) [26], was hybridized against the HQR viral DNA. First, printed slides were hydrated for 30 min at 42°C, then air-dry at room temperature to homogenize the spot size. Next, the slides were washed for 2 min with 2X SSC + 0.1% SDS, followed by a second 2 min wash with 2X SSC. Subsequently, they were denatured in boiling water for 2 min and immediately cooled in ethanol at −20°C for another 2 min. After drying in a microarray high-speed centrifuge (MHC, Arrayit Corp., Sunnyvale, CA, USA), the slides were pre-hybridized for 1 h at 42°C with pre-hybridization solution (containing 25% formamide, 3X SSC, 0.3% SDS, and 100 ng/ml herring DNA) in an Arrayit hybridization cassette. For hybridization, 10 nanograms from the HQR viral DNA were amplified, using the GenomiPhi DNA Amplification Kit (Amersham), sheared for 30 s at 70% amplitude using the Branson digital 450 sonicator, and then labeled using Klenow enzyme and dCTP-Cy3 for 1 h at 37°C. After purification and quantification, around 60 pmol of Cy5-labeled DNA were mixed in Hyblt 2 hybridization solution (Arrayit Corp., Sunnyvale, CA, USA) and incubated overnight at 50°C against the virochip in duplicate. Following hybridization, the slides were washed twice with 0.2X SSC + 0.1% SDS, followed by a final wash in 0.2X SSC for 10 minutes, rinsed in 0.05X SSC for 10 s, and dried in the MHC centrifuge. The hybridized array was scanned for the Cy5 dye in a GenePix 4100A scanner (Axon Instruments Inc.) and the resulting images were analyzed by quantifying the fluorescence intensity of each spot using the GenePix Pro v.6.0 software (Axon Instruments Inc.).

Twenty-eight triplicated fosmids containing haloviral genomes gave a strong positive hybridization signal against the labeled HQR viral DNA. The corresponding 28 *Escherichia coli* EPI300-T1R clones were then separately grown in 5 ml of TB medium containing 0.2% maltose, 12.5 μg/ml of chloramphenicol, and the CopyControl Induction Solution (Epicentre). Twenty-five fosmids were successfully extracted using the FosmidMAX DNA Purification Kit (Epicentre), and sequenced at FISABIO (Valencia, Spain) using a MiSeq System (Illumina) (2x300 bp).

### Virus fluorescence *in situ* hybridization

Nucleotide sequences of three viral conserved genes in the set of sequenced fosmids (genes coding for the portal protein, the major capsid protein, and a scaffolding protease), were aligned with the Geneious R11.1 software [36] to design eight specific primer pairs: P1g1f (5’-CATGAGATCACACAATTCAG-3′), P1g1r (5′-GTAAAGTCAAARTTCTCTGC-3′), P2g1f (5′-ACAATGGAGATTGATGACC-3′), P2g1r (5′-CAAAGTCTCCCTCAACAT-3′), P3g2f (5′-CCAGAACAAGAGGCAGA-3′), P3g2r (5′-GCATCTCGTGCAATCTTY-3′), P4g2f (5′-AGCGKCAGATTGAGTATGT-3′), P4g2r (5′-GTGACAAAACTATCTGRGAT-3′), P5g2f (5′-AAACAGTTGGGGATTCGAT-3′), P5g2r (5′-TTGCTGCTGCTCGYTCT-3′), P6g3f (5′-CTCRGTTGAAATGGCAGA-3′), P6g3r (5′-TGCATCACCATCGGKTTT-3′), P7g3f (5′-GGACAGAYCAAAAATCTCG-3′), P7g3r (5′-GCAGACTGCATATTYTCATC-3′), P8g3f (5′-CCCAAAAGACGCTTATGAA-3′), and P8g3r (5′-CCATCATCATATCTCGCAT-3′). Five nanograms of CR30 DNA were used as the template for the PCR amplification of the corresponding amplicons, with estimated sizes between 183 and 524 base pairs. PCR conditions were as follows: (i) an initial denaturing step at 94°C for 3 minutes, (ii) 30 cycles of denaturing (94°C, 30 s), annealing (58°C, 1 minute), and extension (72°C, 2 minutes), (iii) and a final extension step at 72°C for 30 minutes. Each PCR mixture contained 5 μl of 10X reaction buffer, 1.5 μl of MgCl_2_ 50 mM, 1.0 μl of dNTPs 10 mM, 2.5 μl of each primer at 10 μM, one unit of *Taq* polymerase (5 U/μl, Invitrogen), and sterile ultrapure water up to 50 μl. The eight amplification products were checked by gel electrophoresis, purified with the QIAquick PCR Purification Kit (Qiagen), and a fraction was sequenced in the STAB vida service (Portugal). Obtained sequences were confirmed to be specific for the group of the target haloviral genomes (cluster 1 of eHPs; [25]) by BLASTn analysis against the NCBI database [37]. The remaining purified PCR products were pooled, precipitated, resuspended in 20 μl of the labeling buffer supplied in the ULYSIS Nucleic Acid Labeling Kit (ThermoFisher Sci.), and quantified using Qubit (Invitrogen). Then, 580 ng of DNA were labeled with 15 μl of AlexaFluor 594, by using the abovementioned kit, and purified with Micro Bio-Spin Columns with Bio-Gel P-30 (Bio-Rad) to obtain the virusFISH probes.

For the virusFISH hybridization, a pure culture of *Hqr. walsbyi* (as negative control) and four hypersaline water samples from Bras del Port salterns taken in April 2019 (intermediate salinity ponds CM2 and CO71, with 22% salinity; and CR30 and CR41 crystallizers, with 37% salinity) were fixed with formaldehyde (7% final concentration, 4°C, 16 h), washed with 10 volumes of 1X PBS (phosphate-buffered saline: 137 mM NaCl, 2.7 mM KCl, 10 mM Na_2_HPO_4_, 2 mM KH_2_PO_4_, pH 7.4) and filtered by 0.22 μm GTTP filters (Millipore). Filters were placed in a humid chamber, incubated for 15 minutes with a solution of lysozyme (10 mg/ml), washed with ultrapure water, and dried with absolute ethanol. The labeled probes were mixed with hybridization buffer (35% formamide, 5X SSC buffer, 20% dextran sulfate, 0.1% SDS, 20 mM EDTA, 0.25 μg/ml of salmon sperm DNA, 0.25 μg/ml of yeast RNA, and 1% of blocking agent) at a final concentration of 496 μM. Hybridizations were carried out at 85°C (for 40 minutes) and 46°C (for 2 h) in a 35% formamide-humid chamber. Hybridized filters were subsequently incubated with washing buffer (70 mM NaCl, 20 mM Tris–HCl pH 8.0, 50 mM EDTA pH 8.0, and 0.01% SDS; 15 minutes at 48°C), 1X PBS (20 minutes at room temperature) and ultrapure water (1 minute at room temperature), and then dried with absolute ethanol (38, with modifications). Finally, filters were stained with DAPI and observed in a confocal laser-scanning microscope (Leica, type TCS-SP2; Vashaw Scientific Inc., Norcross, GA, USA). Eight microphotographs per sample were taken for the quantification of the total prokaryote community, the “square” cells, and those cells hybridised with the AlexaFluor 594-probe.

### Single-cell genomics, hybridization, and fosmid 4G12 purification

Fifty microliters of the CR30jun11 water sample were used for single-cell sorting, whole-genome amplification, PCR screening of 16S rRNA genes, and subsequent sequencing of the PCR products at the Bigelow Laboratory of Single Cell Genomics Center as in Gomariz et al. [39]. Four micrograms of five single-amplified genomes (SAGs) affiliated to *Hqr. walsbyi* were sheared by sonication and labelled with Cy3 as described above. Around 50 pmol of each of the five Cy3-labelled targets were independently hybridized against the abovementioned virochip, which was subsequently scanned and analyzed. DNA from fosmid 4G12, which yielded a positive hybridization signal against the labeled DNA from the *Haloquadratum* SAG AB577-A23, was extracted from its *E. coli* clone as described above. Both the fosmid and the SAG were sequenced at FISABIO (Valencia, Spain) using a MiSeq System (Illumina) (2x300 bp).

### Counts and metagenomes from other Bras del Port samples

One liter of brine from each of five different ponds of Bras del Port salterns [two intermediate-salinity ponds (CM1 and CM2), a brine concentrator (CCAB), and two crystallizers (CR30 and CR41)] was collected on February, July, and November 2014, February and May 2015, and September and December 2016, and immediately processed in the laboratory. Salinity was measured *in situ* with a hand refractometer (Eclipse). DAPI and Sybr-Gold staining for cells and VLP counts, respectively, were carried out, while *Archaea* and *Bacteria* were identified by fluorescence *in situ* hybridization (FISH), as described in [34].

Brines were also filtered through 0.2 μm Durapore filters (Millipore) to collect the cell biomass. Total DNA was then extracted from the filters with the RNeasy PowerSoil DNA Elution Kit (QIAGEN), following the manufacturer’s recommendations. The filtered volume, containing the viruses, was concentrated, and the viral DNA extracted as described above. Sequencing of cell and viral DNAs was performed using the mentioned MiSeq System. Additionally, the viral fraction of CR30Feb14 was cloned in fosmids, and genomes from two clones (eHqrV-58 and eHqrV-70) were sequenced as described above.

### Bioinformatic analyses

All sequencing reads were quality assessed and trimmed using Trimmomatic [40]. For viromes, Nonpareil [41] was used to estimate the community coverage and diversity with default parameters. De novo assemblies of trimmed reads were generated using both SPAdes (for fosmids and the SAG AB577-A23) or metaSPAdes (for HQR-V) [42].

HQR-V reads and contigs were initially analyzed using Kaiju [43], and fosmid-derived genomes analyzed by BLASTn alignments against the NCBI database and VIRFAM [44]. VIRIDIC [45] was used to compute pairwise intergenomic distances/similarities among the fosmid-derived and other haloviral genomes. The phylogenetic position of “Haloquadravirinae” members was inferred by using the VipTree software [46]. Proto-spacers were initially identified by using the CRISPRs web server at https://crispr.i2bc.paris-saclay.fr/, and manually confirmed using the Geneious software [36]. Prediction of open reading frames (ORFs) was performed with VirClust [47], which was also used for functional annotation and ORF clustering. The Geneious software (see above) was used to align the gene sequences of fosmid-derived viral genomes to obtain a consensus core genome.

For fragment recruitment analyses, BLASTn comparisons between the “databases” (core genome of “Haloquadravirinae”, viral genome sequence in fosmid 4G12 and CR30 viral contigs from [24]) and the “queries” (virome reads from Bras del Port salterns, HQR-V, and other hypersaline environments) were performed by using a minimum query coverage of 70% and the “besthit” option from the enveomics collection [48]. For recruitments against CR30nov14 and CR30sep16 metagenomes, the viral reads in the metagenomes were first extracted by recruitment against their corresponding viromes. To estimate the abundance of a given viral population, only recruited reads with nucleotide identities above 80% were considered (see the Results and Discussion section). In the case of “Haloquadraviriniae” members, recruitments were calculated against the core genome, and also normalized by the average genome length according to the sequencing depth of the core genes. In the case of genus-level recruitments, genus-specific genes were used. Recruitment plots were drawn with enve.recplot2 in R [48].

## Results and discussion

### Analysis of a *Haloquadratum*-targeted virome

As a first approach to enrich the viruses putatively infecting *Haloquadratum*, a co-culture of the *Hqr. walsbyi* strains HBSQ001 and C23^T^ [15, 16], with 3.8 × 10^5^ cells/ml, was incubated with the natural viral assemblage of a CR30 crystallizer sample (CR30feb14, with 4.9 × 10^8^ VLP/ml). After 19 days of incubation, the abundance of VLPs in the enrichment culture (HQR sample) decreased to 2.1 × 10^8^ VLP/ml. However, we cannot rule out the possibility that some viral replication occurred, potentially replacing a fraction of the original virus population that had decayed. TEM analysis of the HQR viral assemblage post-incubation revealed a notable shift in morphology: spindle-shaped viruses, which accounted for 39% of the original CR30feb14 viral community [35], increased in relative abundance to 68% in the HQR sample.

The enriched virome (HQR-V) was subsequently obtained, and more than 95% of the reads were classified as either “viral” or “unclassified” according to the Kaiju database. The assembly revealed that 93% of the viral contigs were shorter than 1 kb. Among these, approximately half displayed low-GC content (≤50%), suggesting they likely originated from *Haloquadratum* viruses, consistent with the 48% GC content of *Hqr. walsbyi* [21]. Only five contigs with intermediate or high GC content exceeded 10 kb in length (Suppl. Fig. S1). Those with GC content above 65% were attributed to viruses infecting high-GC halophilic hosts present in the original viral community. One contig (vContig01; Suppl. Table S1) exhibited an intermediate GC content of 56.3% and accounted for almost 10% of the total HQR-V reads, despite being a rare component in the original CR30feb14 virome (only 0.004% of the reads). Although viral GC content typically correlates with the genomic GC of their hosts [49], some intermediate-GC viral contigs obtained through shotgun cloning from the CR30 crystallizer [24] were identified as *Hqr. walsbyi* C23^T^ viruses based on CRISPR spacers identity [21]. Therefore, it is plausible that vContig01 was propagated in HQR using one of the *Hqr. walsbyi* strains as its host. Additionally, vContig01 encoded a predicted protein that shared 42% identity with the gp32 of the spindle-shaped virus His1 and lacked the typical genes associated with tailed viruses. These genetic characteristics (Suppl. Fig. S1), combined with the predominance of spindle-shaped viruses in the HQR viral assemblage, led us to hypothesize that vContig01 could belong to a virus of this morphotype. However, we cannot exclude the possibility that this virus may have propagated in a contaminating host present in the enrichment or simply persisted in HQR due to the high stability of its virions, which could explain its increased relative abundance.

As previously mentioned, the HQR-V produced very short contigs with low-GC content. One potential bias in the assembly could stem from the presence of abundant and microdiverse co-occurring genomes within a given viral community, which complicates the reconstruction of longer sequences [50–52]. To further investigate these unidentified low-GC sequences, viral DNA from HQR-V was hybridized to a virochip containing 364 immobilized haloviral genomes from a previous CR30 crystallizer sample (CR30jun11), which likely harbors an unknown fraction of *Haloquadratum* viruses. This virochip, in combination with single-cell genomics, previously allowed us to directly link an uncultured halovirus with its nanohaloarchaeal host [26]. After the hybridization, 28 immobilized viral genomes, which had previously been cloned in fosmids, gave a positive signal with the HQR-V DNA (Suppl. Fig. S2), indicating that those viruses were present in the HQR viral assemblage.

### Several viruses putatively infecting *Haloquadratum* are distant members of the *Haloferuviridae* family

Twenty-five out of the abovementioned 28 viral genomes were successfully sequenced. BLASTn searches showed good matches between them and the cluster 1 of eHPs, uncultured viruses previously predicted to infect *Hqr. walsbyi* and classified as members of the *Caudoviricetes* class (tailed dsDNA viruses) [25]. Although the fosmid-based strategy could be biased towards the preferential cloning of *Caudoviricetes* [25], it is well documented that tailed haloviruses are present in saturated brines, where they can reach up to 30% of the total VLPs [14, 53]. Nineteen out of the 25 sequenced genomes were terminally redundant and then selected for further analyses for which complete genomes are needed. These 19 genomes were homogeneous in size (33.8 ± 1.0 kb), %GC (44.4 ± 0.4%) and number of predicted ORFs (44.9 ± 3.3) (Suppl. Table S1). To further explore this group of viruses, a more comprehensive study was conducted with the 19 abovementioned genomes (from 2011), four eHPs from the mentioned cluster 1 (eHP-E5, from 2007; and eHP-22, eHP-24, and eHP-37, from 2008; [25]), and 2 genomes from CR30feb2014, which also belonged to the same group by BLASTn (Suppl. Table S1), making up a final dataset of 25 complete genomes. Putative proto-spacers against the *Hqr. walsbyi* C23^T^ CRISPR systems were detected in 19 out of the 25 genomes (Suppl. Fig. S3), as previously occurred for some eHPs in cluster 1 [25], pointing to the square archeon as the putative host. Most proto-spacers harbored the 3’ PAMs (proto-spacer adjacent motifs) necessary for an effective interference reaction in type I CRISPR systems of halophilic archaea [25, 54].

The 25 complete genomes, which we will refer to as eHqrVs, from environmental *Haloquadratum*  viruses, formed a monophyletic group close to the tailed dsDNA haloviruses of the family *Haloferuviridae*, siphoviruses which infect *Halorubrum* (HRTV-4, HRTV-29) and *Haloferax* (HFTV-1) [55] (Suppl. Fig. S4). Indeed, VIRFAM [44] classified eHqrVs as siphoviruses based on the organization of their head–neck-tail modules. *Haloferuviridae* members and eHqrVs shared 10 ORFs, constituting ~15% of each haloferuvirus and ~22% of each eHqrV genome. According to Liu and co-workers, which pointed that archaeal tailed viruses of the same family share 20% to 50% of homologous proteins [55], eHqrVs could be distant members of *Haloferuviridae*, for which we propose the creation of the subfamily “Haloquadravirinae” (or haloquadraviruses) (virus subfamilies are created when two or more discrete genera are related below the family level; see below) [56].

**Figure 1:**
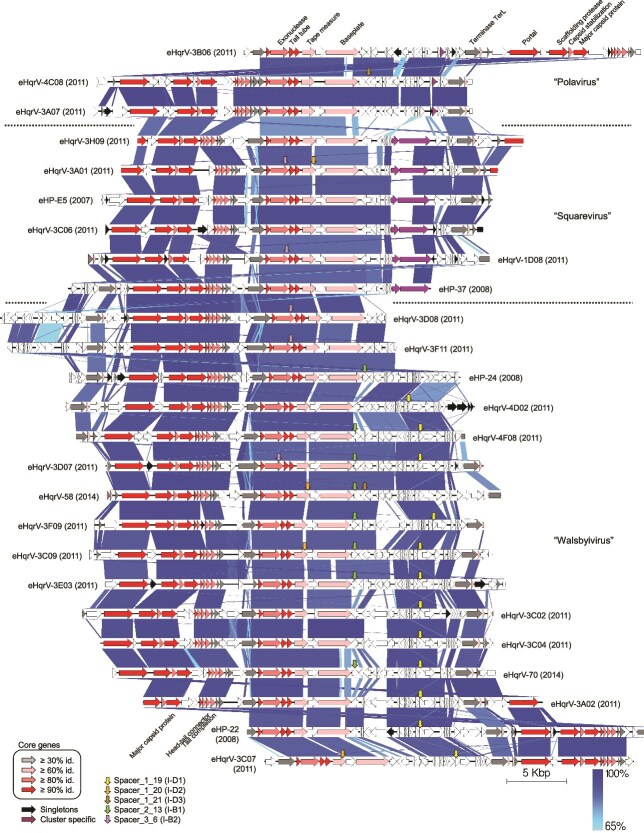
Alignment of the 25 “Haloquadravirinae” complete genomes, retrieved from the CR30 crystallizer in the period 2007–2014, and grouped by genus (“Polavirus”, “Squarevirus” and “Walsbyivirus”). Predicted ORFs are indicated by horizontal arrows. Core genes are coloured according to their amino acid identities (grey, pink, red), whereas genus-specific genes and singletons are coloured in purple and black, respectively. Vertical arrows indicate the genome position of predicted proto-spacers against the *Hqr. walsbyi* C23^T^ CRISPR systems (nomenclature as in 21 and 25). Representation is based on Easyfig (blue intensities are related to the identity percentages in the alignment as shown in the bottom right gradient key) [58].

Haloquadraviruses presented intergenomic similarities between 43.7 and 86.5% (Suppl. Fig. S5), and formed three well-defined clusters in the proteomic tree (Suppl. Fig. S4). The three genomes in cluster A showed intergenomic similarities between 67.1 and 80.9%, and shared 80% of their genes; cluster B grouped 6 genomes with similarities between 67.7 and 83.6%, and 73% of genes shared; and cluster C contained the remaining 15 genomes, with intergenomic similaritiy values from 63.4 to 86.5%, and 65% of genes shared. The current criterion of the ICTV’s Bacterial and Archaeal Viruses Subcommittee (BAVS) defines viral species and genera as cohesive groups of viruses sharing ≥95% and ≥70% of intergenomic similarity, respectively [57]. Also, members of the same archaeal tailed viral genus typically share more than 60% of their proteins [55]. Accordingly, clusters A, B and C might represent three new genera (with some ambiguously classified genomes), for which we propose, respectively, the following names: “Polavirus”, “Squarevirus”, and “Walsbyivirus” (Fig. 1). Every eHqrV genome within each genus might represent a different viral species according to their intergenomic similarity values, below 95%.

The three proposed genera shared a core genome consisting of 20 orthologous genes, most likely responsible for the hybridization signal against the virochip. Core genes were grouped in modules separated by hypervariable regions (Fig. 1), which would have hindered the assembly of these genomes in the enriched virome HQR-V. Core genes constituted around a half of the ORFs in each genome and included four virus hallmark genes (the major capsid protein, the portal protein, an exonuclease and the large subunit of the terminase) and 16 ORFs coding for conserved hypothetical proteins (CHPs), six of them with a putative structural role (Suppl. Table S2). The N-terminus of the largest CHP matched with tail-baseplate viral proteins and its C-terminus harbored a glycosyl hydrolase domain, probably responsible for the degradation of the host cell wall or its extracellular polymeric substance. This CHP might thus be involved in virus-host recognition. Fragment recruitment analysis of the “Haloquadravirinae” core genome against Bras del Port viromes (see below) showed that this C-terminal region was highly variable (Suppl. Fig. S6), as expected for proteins involved in host recognition. Indeed, the variations of this viral protein might mirror the high heterogeneity of the *Hqr. walsbyi* surface [59], the first-line strategy of viral defense in the square archaeon [60].

The pan-genome of “Haloquadravirinae” codes for 113 different proteins so far (Suppl. Table S2), with no genetic markers found that could indicate a lysogenic potential (such as integrases or systems for synchronous replication and partition, such as the Par system of bacteriophage P1; 61). Genera-specific genes were located within the larger hypervariable region containing the largest number of protein-coding genes with no predicted function (Fig. 1), whereas genes coding for DNA methyltransferases were the most frequently found in the flexible genome, accounting for up to 39% of the genes with a predicted function. This fact points to DNA modification as the preferred mechanism for haloquadraviruses to evade the host defense systems. Auxiliary metabolic genes (host genes present in viruses which modulate the host metabolism during the infection) were also detected in some genomes, such as the phosphoadenosine phosphosulfate reductase (PAPS reductase), previously detected in other haloviruses [62] and thought to facilitate sulfate assimilation, and a transporter for precursors of the nucleoside queuosine (Q). Although queuosine is only found in members of *Bacteria* and *Eukarya*, its archaeal analog, the archaeosine (G^+^), is also synthesized from the same precursors. Both queuosine and archaeosine posttranscriptionally modify the tRNAs, influencing translation, tRNA structure and stability, and regulatory events [63]. Moreover, the archaeosine was recently detected in the genome of the Enterobacteria phage 9 g and it was supposed to protect the viral DNA against the host restriction enzymes [64]. Genes for the modification and synthesis of queuosine/archaeosine precursors have also been identified in other haloarchaeal viruses [65].

### Visualization and quantification of “Haloquadravirinae”-infected cells in nature

In order to corroborate that *Haloquadratum* is the host for haloquadraviruses and to quantify the percentage of infected *Haloquadratum* cells in nature, the virusFISH technique was used [32]. As far as we know, virusFISH has only been applied to assign virus-host pairs in natural samples in biofilms from sulfidic springs [66, 67] and sponge tissues [68].

**Figure 2:**
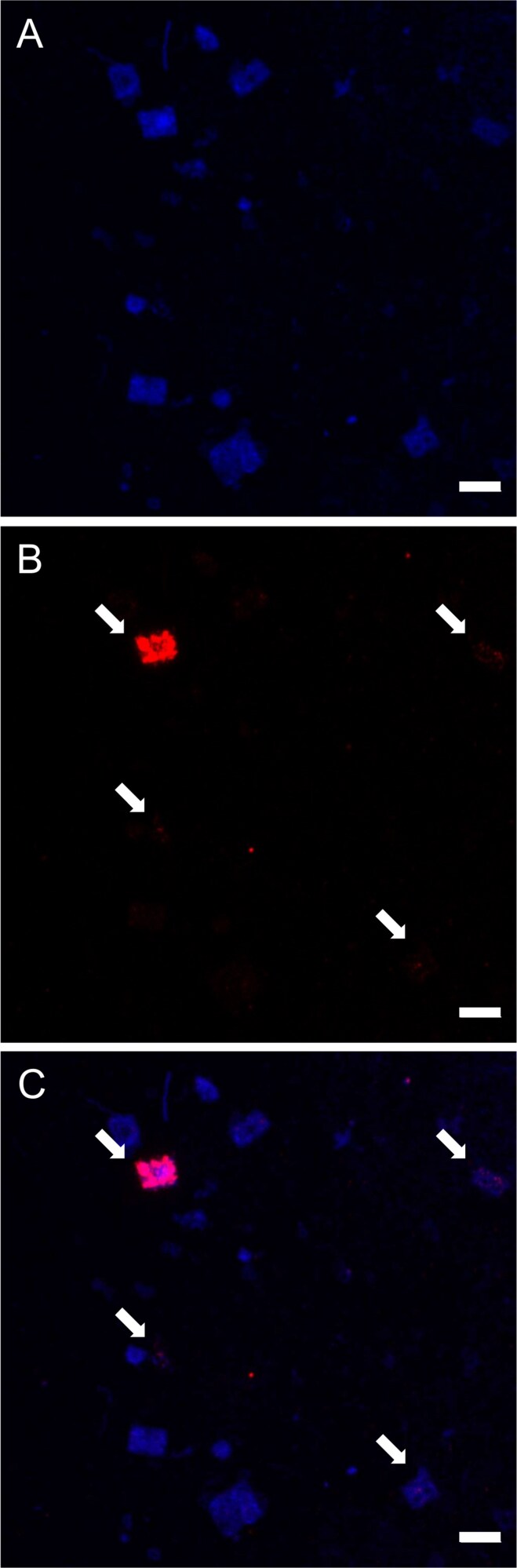
Confocal laser scanning microscopy images of the virusFISH for haloquadraviruses. A. DAPI staining of a CR30apr17 water sample, where square cells are clearly observed. B. Fluorescence of the hybridized AlexaFluor 594-labelled probes for “Haloquadravirinae”. C. Combined DAPI-virusFISH image. White arrows indicate some infected square cells. Scale bar: 5 µm.

Specific probes for haloquadraviruses, targeting three of the most conserved core genes (those coding for the portal protein, the major capsid protein, and the putative scaffolding protease), were designed and hybridized against cells from several Bras del Port brine samples with different salinities and concentration of square cells (Fig. 2; Table 1). The proportion of square cells infected by haloquadraviruses with respect to the total square cells was ~10 percentage points higher at intermediate salinities than in those samples with salinities around 37% (Table 1). This result might suggest that haloquadraviruses are less infectious, and/or their hosts less susceptible to infection, at salinities close to saturation. Nevertheless, the number of infected cells may not directly correlate with viral lysis events. Although no genetic markers indicating lysogeny were identified, we cannot exclude the possibility that haloquadraviruses could establish a pseudolysogenic relationship with their hosts. Pseudolysogeny has been observed in viruses infecting the extreme halophilic bacterium *Salinibacter ruber* [69], where viruses persist episomally in the cytoplasm as a strategy to protect the cell from subsequent infection and lysis under conditions of high multiplicity of infection [69]. This mechanism is consistent with the high virus-to-cell ratios observed in hypersaline aquatic environments [14, 23].

### Haloquadraviruses are highly abundant in hypersaline environments

**Figure 3:**
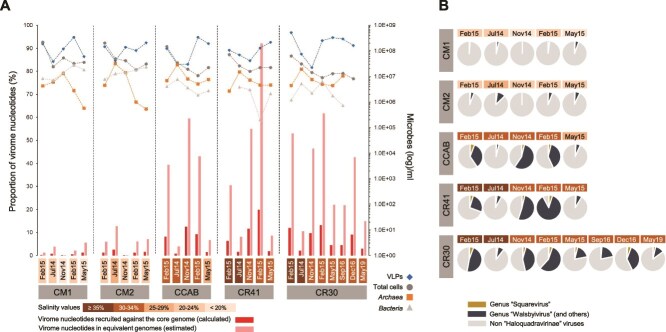
A. Relative abundance of “Haloquadravirinae” in a set of 28 viral metagenomes from Bras del Port salterns, spanning the period 2014–2019. Red bars represent the percentage of viromic nucleotides recruited by five “Haloquadravirinae” core genes, whereas pink bars refer to the estimated percentage of nucleotides recruited after normalization of the core genome sequencing depth by the average genome length of haloquadraviruses. The dynamics of VLP, total cells, *Archaea* and *Bacteria* per ml of brine sample are also indicated. B. Estimated percentages of “Haloquadravirinae” genera compared with other haloviruses in the studied system.

Five core genes of haloquadraviruses (including the major capsid protein, the portal protein, a putative tail protein, the scaffolding protease, and a hypothetical protein) were used to estimate the contribution of this group of viruses in the enriched *Haloquadratum* virome (HQR-V) as well as in a set of viromes from Bras del Port salterns spanning from 2014 to 2019 (this work; [69]). Haloquadraviruses recruited 5.6% of the total nucleotides in HQR-V, and their relative abundances in the salterns ranged from ~0.1% to up to 92.2% (Fig. 3A; Suppl. Table S3). Specific genes in genera “Polavirus” and “Squarevirus” were also used to calculate their proportions (Fig. 3B; Suppl. Table S4). The third genus, “Walsbyivirus”, lacked a specific gene (Fig. 1), and thus the remaining reads, which were the most abundant, would correspond to this genus, though we cannot rule out the possibility that other genera, not detected in our set of analyzed genomes, are also contributing to the abundances.

As expected, the highest proportion of haloquadraviruses (in percentage of recruited nucleotides) corresponded to ponds with the highest salinity values (CCAB, CR30, and CR41), where *Haloquadratum* dominates the microbial community. In these ponds, the archaeal maxima observed in July were accompanied by the lowest relative abundances of haloquadraviruses (Fig. 3A, 3B). Although this observation might fit with the classical “Kill-the-Winner” dynamics, if we consider that most archaea at salt-saturated conditions are very likely members of *Haloquadratum*, the lack of data on *Haloquadratum* numbers makes us cautious about proposing a co-variation model. Also, at intermediate salinities (CM1 and CM2 ponds), the highest proportion of haloquadraviruses corresponded with the archaeal maxima (Fig. 3A), although in these ponds, the archaeal community is not dominated by the square archaeon [11]. The dynamics of haloquadraviruses along the salinity gradient might then rely on the succession of host ecotypes with different temperature and/or salinity optima. Indeed, Viver and co-workers identified *Hqr. walsbyi* metagenome-assembled genomes whose relative abundances varied along salinity transitions, with differences in their average nucleotide identity (ANI) values and gene content [70]. More recently, a metatranscriptomic study in the CR30 crystallizer revealed differences in the gene expression patterns of *Haloquadratum* and the cluster 1 of “environmental halophages” (“Haloquadravirinae” members) between summer and winter [71].

The relative abundances of haloquadraviruses were also estimated in two Bras del Port cellular metagenomes, for which their corresponding viromes were available (CR30nov14 and CR30sep16 samples), to unveil their activity at the time of sampling. Relative abundances were 3.6% and 0.3% of the total viral nucleotides in both metagenomes, respectively, with ratios between 13 and 75 times higher in the extracellular fraction (viromes) than inside the cells (metagenomes). In the absence of more information regarding other active members of the haloviral community, this fact could be explained by the presence of a significant number of extracellular virions from previous infections, which remain highly stable under salt saturation conditions but may have lost their infectivity against current hosts. Additionally, as discussed earlier, the high virus-to-cell ratio observed in these environments might also induce a protective effect against viral lysis within the host population, possibly through mechanisms such as pseudolysogeny [69]. Whatever the scenario, our observations highlight that only the analysis of viromes, without considering the cell fraction, can led to misinterpretations about the meaning of the extracellular virome. A good example of this was the high activity of the uncultured nanohaloarchaeal virus NHV-1, much less abundant than haloquadraviruses in crystallizers, but one of the most active haloviruses unveiled by metatranscriptomics [72].

**Table 1:** Cell counts and percentages of square cells infected by haloquadraviruses in hypersaline samples.

**Sample**	**Salinity**	**Cells/ml (x10** ^**7**^ **)**	**Square cells/ml (x10** ^**7**^ **) (%** ^**a**^)	**Infected square cells/ml (x10** ^**6**^ **), %** ^**b**^
**CM2apr19**	22% (intermediate)	2.5 ± 0.7	0.3 ± 0.1 (10.5%)	1.0 ± 0.04 (37.8%)
**CO71apr19**	22% (intermediate)	3.3 ± 0.5	0.4 ± 0.1 (12.3%)	1.4 ± 0.5 (35.7%)
**CR30apr19**	37% (high)	2.5 ± 0.4	1.4 ± 0.2 (55.6%)	3.7 ± 1.1 (26.8%)
**CR41apr19**	37% (high)	3.1 ± 0.7	1.4 ± 0.4 (43.6%)	3.5 ± 0.9 (25.7%)

a Percentage of square cells with respect to cells/ml (considering the averages).

b Percentage of infected square cells with respect to square cells/ml (considering the averages).

### Other *Haloquadratum* virus-related elements unveiled by single-cell genomics

**Figure 4:**
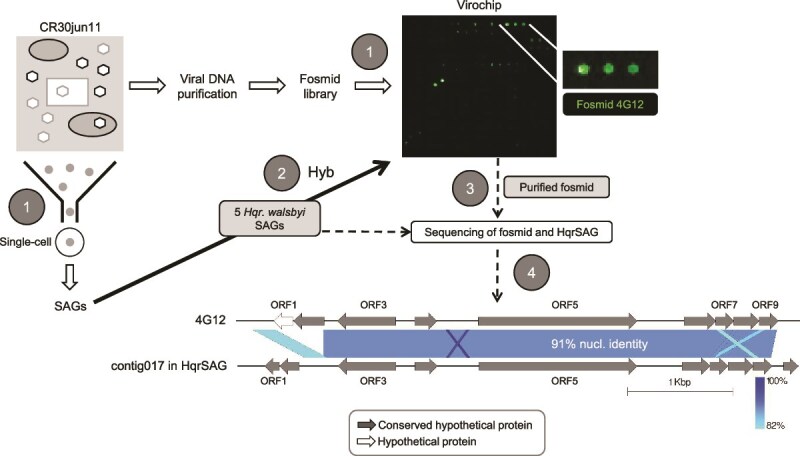
Experimental design (adapted from [26]) used to link uncultured viruses to individual cells. Step 1 shows the isolation of SAGs from a CR30 sample collected in June 2011, alongside the parallel construction of a virochip. Step 2 depicts the separate hybridization of five *Haloquadratum* SAGs against the virochip. Fosmid 4G12 exhibited positive hybridization with SAG AB577-A23 (hereafter referred to as HqrSAG). In Step 3, both fosmid 4G12 and HqrSAG were sequenced, and subsequent analyses confirmed that the fosmid 4G12 insert shared a high sequence identity with one of the contigs from HqrSAG (contig017). Predicted ORFs in both 4G12 and contig017 are marked by arrows and color-coded according to their putative functions. Genome visualizations were generated using Easyfig, where the intensity of blue shading corresponds to the percentage identity in the alignment [58].

In order to directly link viruses with infected *Haloquadratum* cells, we independently hybridized five *Hqr. walsbyi* SAGs from the abovementioned CR30jun11 water sample [26] against the virochip. SAG AB577-A23 yielded positive hybridization signal against fosmid 4G12 (Fig. 4), indicating that the *Hqr. walsbyi* cell from which the SAG originated contained the genetic element cloned in that fosmid at the time of sampling.

The insert of fosmid 4G12 was a concatemer of five units of a small sequence of 5.4 kb, with a GC content of 43% (Suppl. Table S1). We will refer to this sequence as 4G12. The assembly of the SAG produced a contig (contig017) responsible for the hybridization signal between the SAG and the virochip. Contig017 had a size of 5.3 kb, 43% GC and 91% identity with most of the 4G12 sequence (Fig. 4). None of the nine predicted ORFs in 4G12 matched with functionally-assigned viral proteins. However, five gene products (Suppl. Table S5) showed high amino acid identities with hypothetical proteins found in uncultured haloviruses from Spanish salterns [18]. Also, the 4G12 DNA sequence recruited up to 0.28% of the total nucleotides from the set of Bras del Port viromes, frequently along its entire length and, as expected, higher recruitments corresponded to higher salinity ponds (Suppl. Table S6). This indicates that, although we cannot definitively classify the 4G12 sequence as “viral”, this genetic element is consistently present in the extracellular viral fraction of hypersaline environments.

The largest putative ORF of 4G12, with a helix-turn-helix DNA-binding domain (a putative transcriptional regulator), matched with haloarchaeal hypothetical proteins and with the protein 11 from the pleomorphic betapleolipovirus HHPV-3 (*Haloarcula hispanica* pleomorphic virus 3) [73]. This protein also has homologs in the PL6-family plasmids of *Hqr. walsbyi* (protein F3) [22]. As mentioned in the introduction section, the PL6-family plasmids, with lengths between 6.1 and 7.0 kb, have a “strong link” to haloviruses based on similar genes and gene synteny. Thus, all these data point to the existence of a group of mobile genetic elements, with a winged helix-turn-helix DNA-binding protein as the signature marker, which might include 4G12 and the so-called ViPREs (“virus and plasmid related elements”) containing the betapleolipoviruses and the PL6-plasmids [74].

### Concluding remarks

The *Haloquadratum* virosphere currently includes two distinct groups of viruses with differing GC content. Other virus-related sequences, which require further investigation to confirm their host or nature, are not considered at this stage.

The first group, unveiled in this study, builds upon previous information: the cluster 1 of “environmental halophages”, which were *in silico* associated with *Hqr. walsbyi* [25]. This group includes members of the newly proposed subfamily “Haloquadravirinae”, which consists of abundant, low-GC (~44.4%) tailed viruses with a genome size of ~34 kb, and three candidate genera. This genome size corresponds to that of the most prevalent viral genomes found in hypersaline environments, as determined by pulsed-field gel electrophoresis [23, 24]. Haloquadraviruses exhibit stability in their core genome, whereas approximately half of their genomes contain genes associated with the flexible genome of the group. Due to the challenges of culturing *Hqr. walsbyi* in the laboratory, the virusFISH technique applied to natural samples has allowed confirmation that members of the “Haloquadravirinae” subfamily indeed infect the square archaeon. The second group, previously described by Dyall-Smith and colleagues [21], includes viral contigs with intermediate GC content. However, taxonomic information and details about their life strategies remain unknown due to the absence of complete genomes.

The aforementioned viral groups are not only autochthonous to the solar salterns of Bras del Port, but they were also detected in other hypersaline systems where *Haloquadratum* is present, such as the multi-pond solar salterns of San Diego (CA, USA; [73]) and the Balearic Islands (Spain; 75), as well as in natural hypersaline environments like Lake Tyrrell (Australia; 76) (Suppl. Table S7). Thus, the *Haloquadratum* virosphere summarized in this study, which is ubiquitous across high-salinity environments worldwide, broadens the scope of our still limited understanding of archaeal viruses.

## Supplementary Material

Villamor_et_al_11th_July_2025-Supplementary_Figures_wraf149

Villamor_et_al_2025-Supplementary_Tables_wraf149

## Data Availability

The raw sequences of the Bras del Port viromes generated during the current study are available in the European Nucleotide Archive (https://www.ebi.ac.uk/ena/browser/home) with the following run accession numbers: ERR13105013 to ERR13105038. The fosmid-derived sequences analysed in this work study are deposited in the GenBank NCBI database (https://www.ncbi.nlm.nih.gov/) under the accession numbers MT764210 to MT764230 (viral genomes from “Haloquadravirinae”) and PV387967 (sequence 4G12).
