# Supplementary material for: Novel viruses of Haloquadratum walsbyi expand the known archaeal virosphere of hypersaline environments
Source: ISME J. 2025 Jul 17;19(1):wraf149. doi: 10.1093/ismejo/wraf149 (PMC12418951; doi:10.1093/ismejo/wraf149)
Supplement: Villamor_et_al_11th_July_2025-Supplementary_Figures_wraf149 [file villamor_et_al_11th_july_2025-supplementary_figures_wraf149.pdf]

A

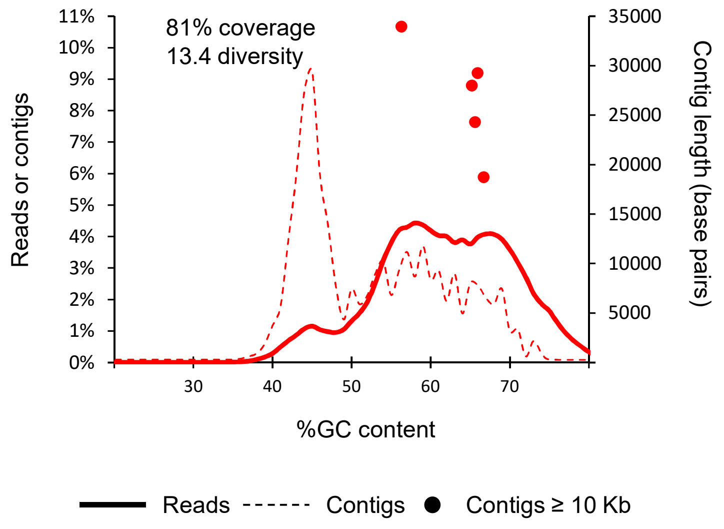

B

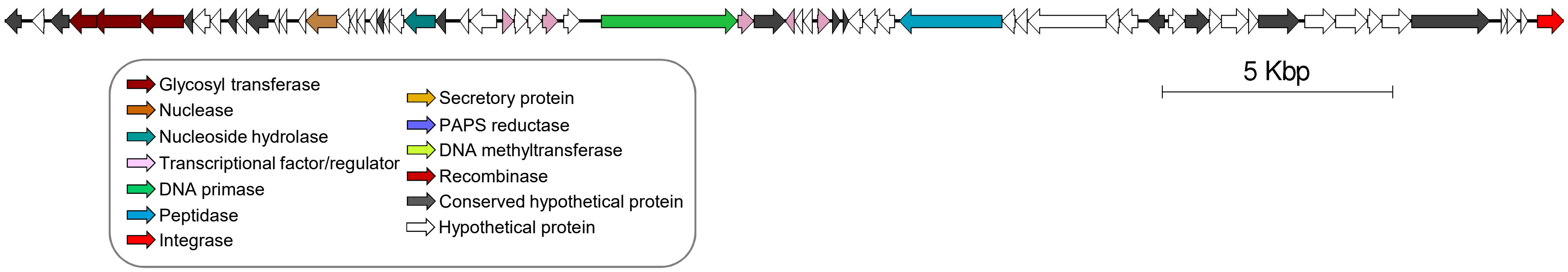

Supplementary Figure S1. A. Left y-axis: Percentages of reads (continuous red line) and contigs (dotted red line) in the HQR virome, according to their GC values (x-axis). Right y-axis: Lenghts of those contigs above 10 kb, according to their GC values (x-axis). Nonpareil parameters (coverage and diversity) are also indicated. **B.** Viral contig vContig01, assembled from the HQR-V. Predicted ORFs are indicated by arrows and coloured according to their putative functions. Representation is based on Easyfig.

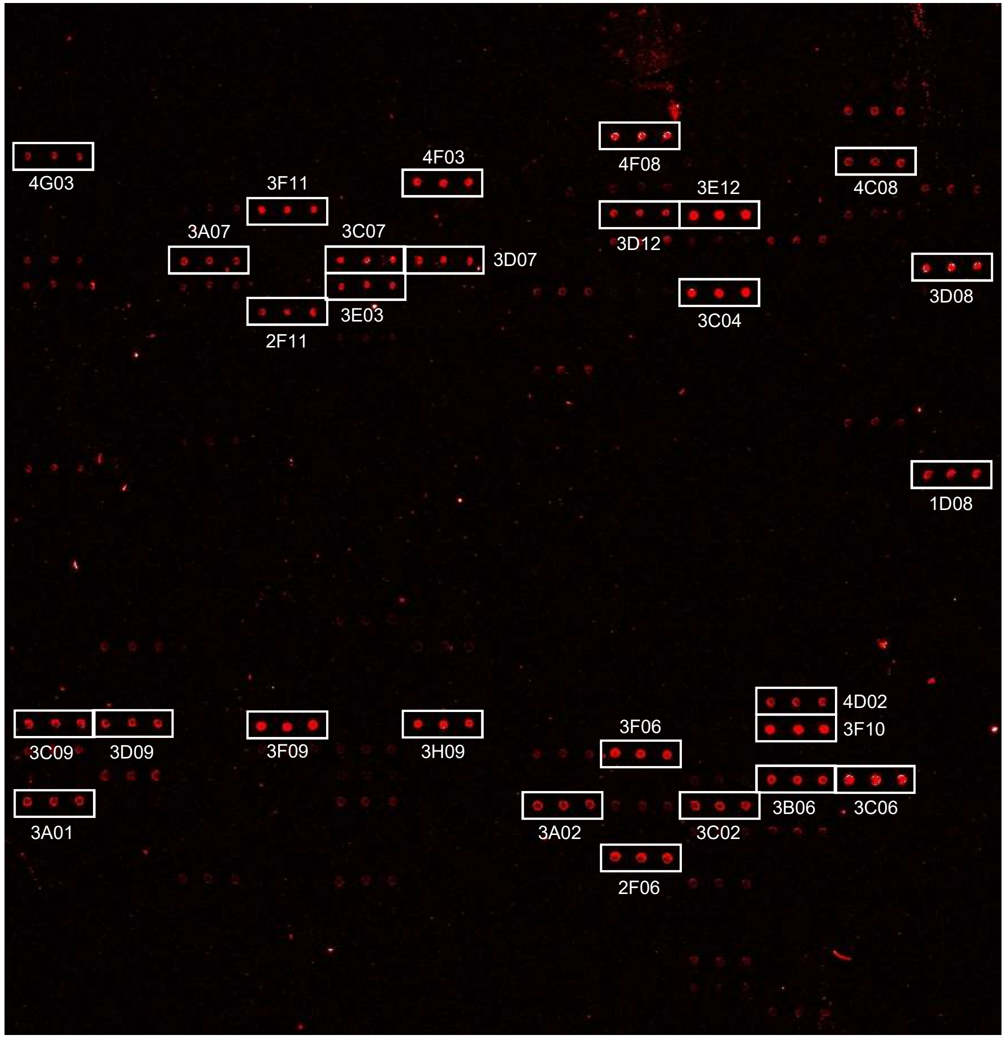

Supplementary figure 2. "Virochip" containing 364 triplicated and immobilized viral fosmid clones from CR30 (Martínez-García et al., 2014) hybridized against the viral DNA from HQR (labelled with Cy5). The squares indicate those fosmids which were selected for sequencing.

		Spacer 19 (CRISPR 1; subtype I-D)			Spacer 20 (CRISPR 2; subtype I-D)			Spacer 21 (CRISPR 1; subtype I-D)		
		ACAGGTAATCTTATCGATATTCGAGAGTTCCGTGCA			AACAGTATTCACTGGGTCCTCTGTCGGAGTCAT			CCTCTACGGCAGTCAACCCTACGCCAGTTGCCCATGT		
**Year**	**EHqrV-1 genome**	**Putative protospacer**	**PAM**	**%ID.**	**Putative protospacer**	**PAM**	**%ID.**	**Putative protospacer**	**PAM**	**%ID.**
2007	eHP-E5									
2008	eHP-22	ACAGGTAATCTTATCGATATTCGAGAGTTCCGTGCA	GGC	100.0						
	eHP-24	ACGGGTCATCTTATTGATATTCGGCATTTCCAGCCA	GGG	75.0				CCTCTACGGCAGTTAGTCCCACGCCGGTAGCCCATGT	TGC	83.8
	eHP-37									
2011	eHqrV-1D08									
	eHqrV-3A01				AACAGTATTCACTGGGTCCTCTGTCGGAGCCAT	CGC	97.0			
	eHqrV-3A02	ACAGGTAATCTTATCGATATTCGAGAGTTCCGTGCA	GGC	100.0						
	eHqrV-3A07							CTTCAACGGCAGTCAACCCAACGCCGGTTGCCCATGT	TGC	89.2
	eHqrV-3B06							CTTCAACGGCAGTCAAACCTACGCCGGTTGCCCATGT	TGC	89.2
	eHqrV-3C02	ACAGGCAACCTTATCGATATTCGAGAGTTCCGTGCA	GGC	94.4						
	eHqrV-3C04	ACAGGTAATCTTATCGATATTCGAGAGTTCCGTGCA	GGC	100.0						
	eHqrV-3C06									
	eHqrV-3C07	ACAGGCAATCTCATCGATATTCGAGAGTTCCGTGCA	GGC	94.4	AACAGTATTCACTGGGTCATCTGTCGGACTCAT	CGC	93.9	CCTCTACGGCAGTTAGTCCCACGCCGGTAGCCCATGT	TGC	83.8
	eHqrV-3C09	ACAGGTAATCTTATCGATATTCGAGAGTTCCGTGCA	GGC	100.0	AACAGTATTCACTGGGTCATCTGTCGGACTCAT	CGC	93.9	CCTCTACGGCAGTTAGTCCCACGCCGGTAGCCCATGT	TGC	83.8
	eHqrV-3D07	ACAGGTAATCTTATCGATATTCGAGAGTTCCGTGCA	GGC	100.0				CCTCTACGGCAGTTAGTCCCACGCCGGTAGCCCATGT	TGC	83.8
	eHqrV-3D08	ACAGGCAACCTTATCGATATTCGAGAGTTCCGTGCA	GGC	94.4	AACAGTATTCACTGGGTCATCTGTCGGACTCAT	CGC	93.9	CCTCTACGGCAGTTAGTCCCACGCCGGTAGCCCATGT	TGC	83.8
	eHqrV-3E03	ACAGGTAATCTTATCGATATTCGAGAGTTCCGTGCA	GGC	100.0				CCTCTACGGCAGTTAGTCCCACGCCGGTAGCCCATGT	TGC	83.8
	eHqrV-3F09	ACAGGTAATCTTATCGATATTCGAGAGTTCCGTGCA	GGC	100.0				CCTCAACAGCAGTTAGTCCAACGCCGGTAGCCCATGT	TGC	78.4
	eHqrV-3F11	ACGGGTCATCTTATTGATATTCGGCATTTCCAGCCA	GGG	75.0				CCTCTACGGCAGTTAGTCCCACGCCGGTAGCCCATGT	TGC	83.8
	eHqrV-3H09									
	eHqrV-4C08							CTTCAACGGCAGTCAACCCAACGCCAGTTGCCCATGT	TGC	91.9
	eHqrV-4D02	ACAGGTAATCTTATCGATATTCGAGAGTTCCGTGCA	GGC	100.0	AACAGTATTTACTGGGTCATCTGTCGGACTCAT	CGC	90.9			
	eHqrV-4F08	ACAGGTAATCTCATCGATATTCGAGAGTTCCGTGCA	GGC	97.2	AACAGTATTTACTGGGTCATCTGTCGGACTCAT	CGC	90.9	CCTCTACGGCAGTTAGTCCCACGCCGGTAGCCCATGT	TGC	83.8
2014	eHqrV-58	ACAGGTAATCTAATCGATATTCGAGAGTTTTCGTGCA	GGG	94.4	AACAGTATTCACTGGGTCATCTGTCGGACTCAT	CGC	93.9	CCTCTACGGCAGTCAACCCTACGCCAGTAGCCCATGT	TGC	97.3
	eHqrV-70	ACAGGTAATCTTATCGATATTCGAGAGTTCCGTGCA	GGC	100.0				CCTCTACGGCAGTTAGACCTACGCCGGTAGCCCATGT	TGC	86.5

		Spacer 13 (CRISPR 2; subtype I-B)			Spacer 6 (CRISPR 3; subtype I-B)		
		TATTGATTATTCGTTTGATTCAAACGTTAATATCG			ATGTTTGTCTTATGGGTCATCATCATATCTCAGGTC		
**Year**	**EHqrV-1 genome**	**Putative protospacer**	**PAM**	**%ID.**	**Putative protospacer**	**PAM**	**%ID.**
2007	eHP-E5				ACGTTGCGCTTATGGGTCATCACCACATATCAGGGA	GAA	75.0
2008	eHP-22				ACGTTGCGCTTATGGGTCATCATCATATCTCTGGGA	GAA	80.6
	eHP-24	TATTGATTATTCGTTTGACTCAAACGTTACCATCG	GAA	91.4	ACGTTGCGCTTATGGGTCATCATCATATCTCTGGGA	GAA	80.6
	eHP-37 (a)				ACGTTGCGCTTATGGGTCATCATCATATCTCAGGTC	GAA	88.9
2011	eHqrV-1D08				ATGTTTGTCTTATGGGTCATCATCATATCTCGGGTA	GAA	94.4
	eHqrV-3A01				ATGTTTGTCTTATGGGTCATCATCATATCTCAGGTC	GAA	100.0
	eHqrV-3A02				ACGTTGCGCTTATGGGTCATCATCATATCTCAGGTA	GAA	86.1
	eHqrV-3A07	TATTAATTACTCGTTTGACTCAAACGTCAGAATTG	ACG	80.0	ACGTTGCGCTTATGGGCCATCATCATATCTCAGGTC	GAA	86.1
	eHqrV-3B06	TATTAATTACTCGTTTGACTCAAACGTCAGAATTG	ACG	80.0	ACGTTGCTCTTATGGGCCATCACCATATCTCAGGTC	GAA	86.1
	eHqrV-3C02				ACGTTGCGCTTATGGGTCATCATCATATCTCAGGTC	GAA	88.9
	eHqrV-3C04				ACGTTGCGCTTATGGGTCATCATCATATCTCAGGTC	GAA	88.9
	eHqrV-3C06				ACGTTGCGCTTATGGGTCATCATCATATCTCAGGTC	GAA	88.9
	eHqrV-3C07	TATTGATTATTCGTTTGACTCGAACGTTACCATCG	GAA	88.6	ACGTTGCGCTTATGGGTCATCATCATATCTCAGGTA	GAA	86.1
	eHqrV-3C09	TATTGATTATTCGTTTGACTCAAACGTTACCATCG	GAA	91.4	ACGTTGCGCTTATGGGGCATCATCATATCTCAGGCA	GAA	80.6
	eHqrV-3D07	TATTGATTATTCGTTTGACTCAAACGTTACCATCG	GAA	91.4	ATGTTTGCCTTATGGGTCATCATCATATCTCTGGGA	GAA	91.7
	eHqrV-3D08	TATTGATTATTCGTTTGACTCGAACGTTACCATCG	GAA	88.6	ACGTTGCGCTTATGGGGCATCATCATATCTCAGGTC	GAA	86.1
	eHqrV-3E03	TATTGATTATTCGTTTGACTCAAACGTTAACATCG	GAA	94.3	ACGTTGCGCTTATGGGTCATCATCATATCTCAGGTC	GAA	88.9
	eHqrV-3F09	TATTGATTATTCGTTTGACTCAAACGTTACCATCG	GAA	91.4	ACGTTGCGCTTATGGGTCATCATCATATCTCAGGTA	GAA	86.1
	eHqrV-3F11	TATTGATTATTCGTTTGACTCGAACGTTACCATCG	GAA	88.6	ATATTTGTCTTATGGGTCATCATCATATCTCAGGCA	GAA	91.7
	eHqrV-3H09				ATGTTGCGCTTATGGGGCATCATCATATCTCTGGTC	GAA	86.1
	eHqrV-4C08				ACGTTGCGCTTATGGGCCATCATCATATCTCAGGTC	GAA	86.1
	eHqrV-4D02				ATGTTGCGCTTATGGGTCATCACCACATATCAGGGA	GAA	77.8
	eHqrV-4F08	TATTGATTATTCGTTTGACTCAAACGTTAACATCG	GAA	94.3	ACGTTGCGCTTATGGGGCATCATCATATCTCTGGTC	GAA	83.3
2014	eHqrV-58	TATTGATTATTCGTTTGACTCAAACGTTACCATCG	GAA	91.4	ACGTTGCGCTTATGGGTCATCATCATATCTCTGGGA	GAA	80.6
	eHqrV-70	TATTGATTATTCGTTTGACTCAAACGTTAACATCG	GAA	94.3	ACGTTGCGCTTATGGGTCATCATCATATCTCTGGGA	GAA	80.6

Supplementary Figure 3. Putative proto-spacers in the set of 25 eHqrV genomes. Variations in the proto-spacer sequence, respecting to the spacer, are marked in yellow. The PAM motifs are marked in orange (dark orange indicates conserved nucleotides). Those proto-spacers matching their corresponding spacers with identities above 90% are marked in blue.

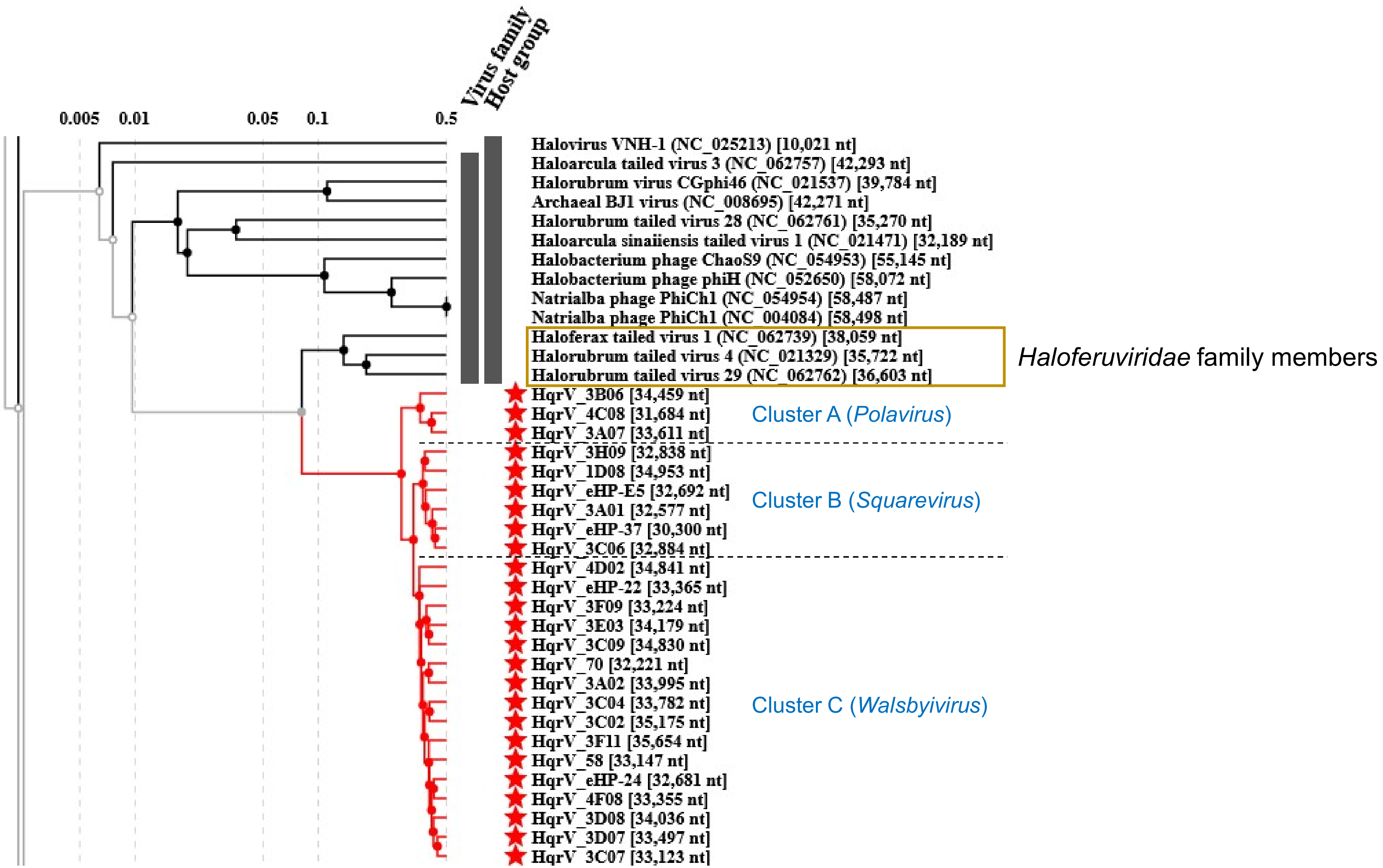

Supplementary figure 4. VipTree analysis of "Haloquadravirinae" members (eHqrVs, marked with red stars) and their position with respect to the *Haloferuviridae* family and other haloviruses. Three clusters of eHqrVs, corresponding to three new genera, are appreciated.

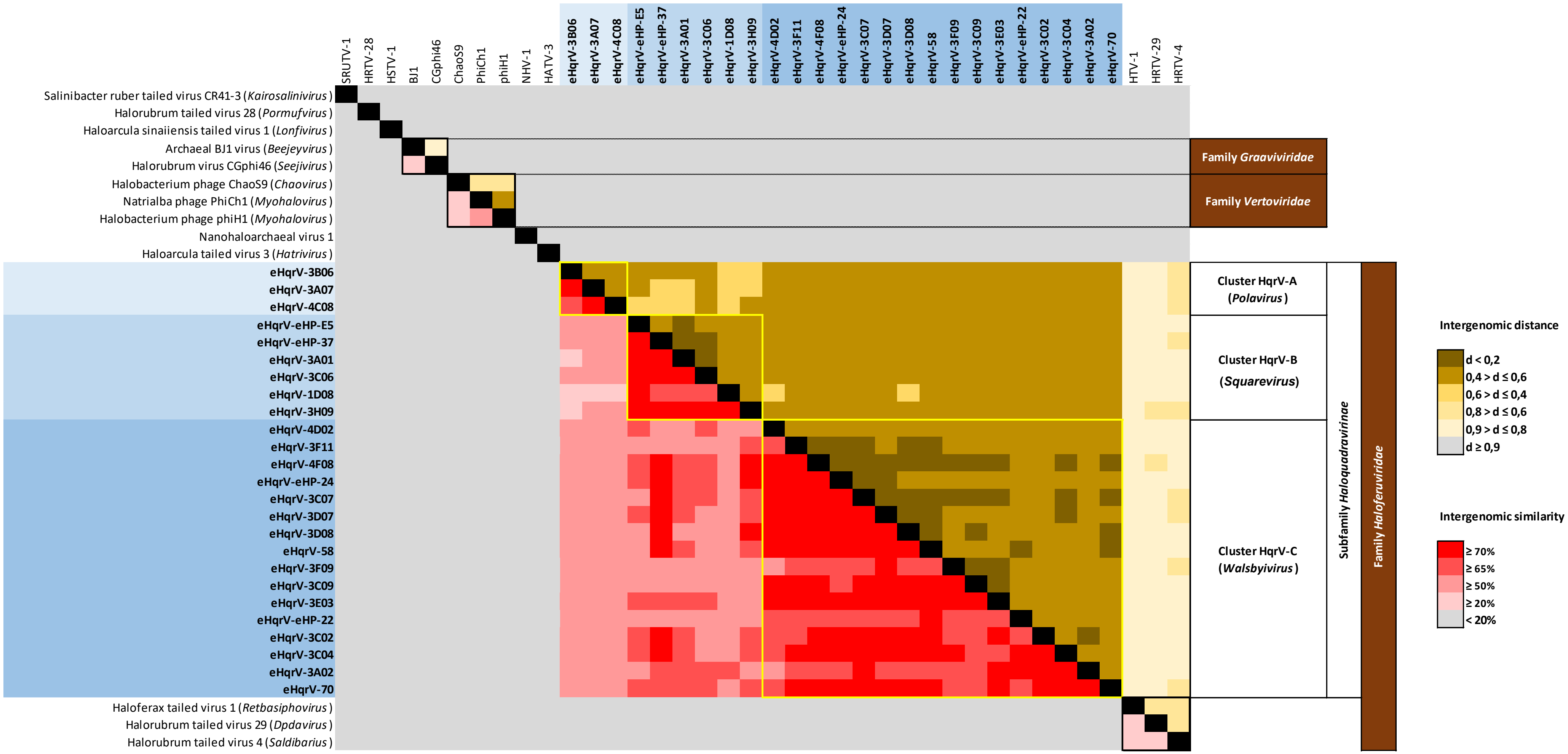

Supplementary figure 5. VirClust-derived matrix of intergenomic similarity values (based on DNA sequences) and intergenomic distances (based on the protein content) among “Haloquadravirinae” and other haloviruses.

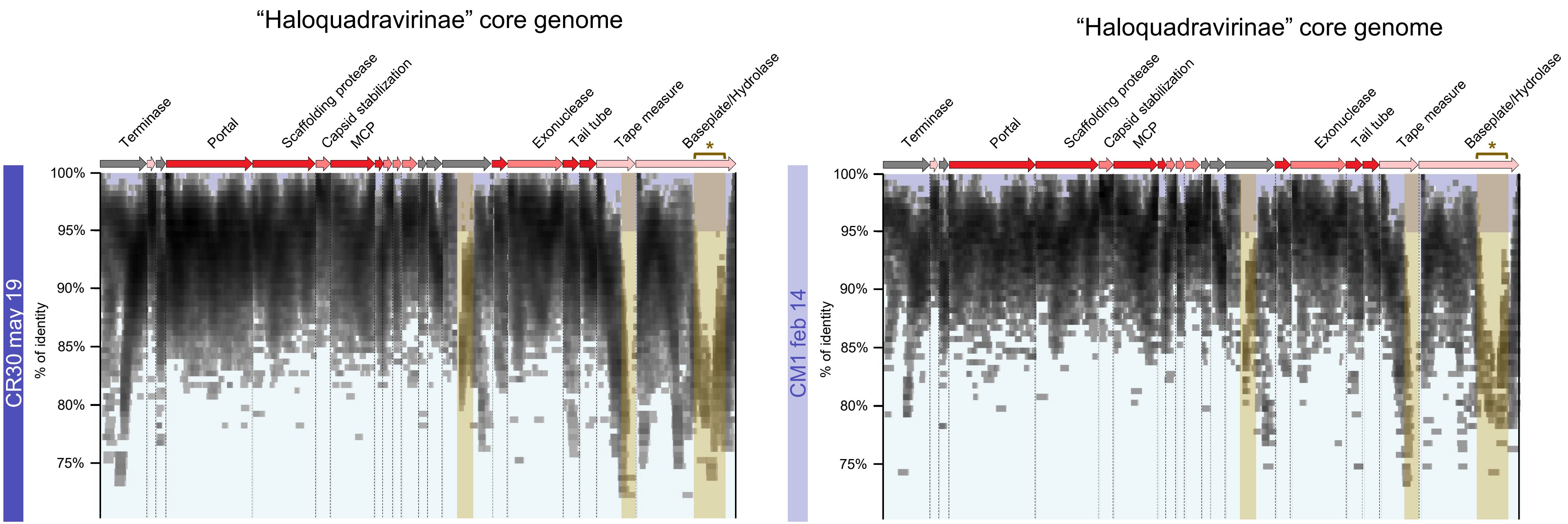

Supplementary figure 6. Fragment recruitment analysis of CR30may19 (left) and CM1feb14 (right) virome reads against the “Haloquadravirinae” core genome. Genes colours as in Figure 1. Hypervariable regions are marked in gold. The asterisk indicates the C-terminal region of a putative baseplate protein where a glycosyl hydrolase domain was predicted.
